# Spatial distribution of marker gene activity in the mouse lung during alveolarization

**DOI:** 10.1016/j.dib.2018.10.150

**Published:** 2018-11-03

**Authors:** M. Cecilia Ljungberg, Mayce Sadi, Yunguan Wang, Bruce J. Aronow, Yan Xu, Rong J. Kao, Ying Liu, Nathan Gaddis, Maryanne E. Ardini-Poleske, Tipparat Umrod, Namasivayam Ambalavanan, Teodora Nicola, Naftali Kaminski, Farida Ahangari, Ryan Sontag, Richard A. Corley, Charles Ansong, James P. Carson

**Affiliations:** a Baylor College of Medicine, Houston, TX, USA; b Jan and Dan Duncan Neurological Research Institute at Texas Children׳s Hospital, Houston, TX, USA; c Texas Advanced Computing Center, The University of Texas at Austin, Austin, TX, USA; d Harvard Medical School, Boston, MA, USA; e Cincinnati Children׳s Hospital Medical Center, Cincinnati, OH, USA; f RTI International, Research Triangle Park, NC, USA; g University of Alabama at Birmingham, Birmingham, AL, USA; h Yale University, New Haven, CN; i Pacific Northwest National Laboratory, Richland, WA, USA

## Abstract

This data is a curated collection of visual images of gene expression patterns from the pre- and post-natal mouse lung, accompanied by associated mRNA probe sequences and RNA-Seq expression profiles. Mammalian lungs undergo significant growth and cellular differentiation before and after the transition to breathing air. Documenting normal lung development is an important step in understanding abnormal lung development, as well as the challenges faced during a preterm birth. Images in this dataset indicate the spatial distribution of mRNA transcripts for over 500 different genes that are active during lung development, as initially determined via RNA-Seq. Images were systematically acquired using high-throughput *in situ* hybridization with non-radioactive digoxigenin-labeled mRNA probes across mouse lungs from developmental time points E16.5, E18.5, P7, and P28. The dataset was produced as part of The Molecular Atlas of Lung Development Program (LungMAP) and is hosted at https://lungmap.net. This manuscript describes the nature of the data and the protocols for generating the dataset.

**Specifications table**

**Table:** 

Subject area	Developmental biology
More specific subject area	Mouse lung development, Genomics, histology
Type of data	Images
How data was acquired	Zeiss AxioScan Microscope
Data format	PNG images, XLS data files
Experimental factors	OCT embedded embryonic torsos and OCT/PBS/sucrose inflated postnatal lungs were frozen and sectioned
Experimental features	High-throughput *in situ* hybridization using non-radioactive digoxigenin-labeled probes
Data source location	Houston, Texas, USA
Data accessibility	Publicly available at https://www.lungmap.net
Related research article	Maryanne E Ardini-Poleske, Robert F Clark, Charles Ansong, James P Carson, Richard A Corley, Gail H Deutsch, James S Hagood, Naftali Kaminski, Thomas J Mariani, Steven S Potter, Gloria S Pryhuber, David Warburton, Jeffrey A Whitsett, Scott M Palmer, Namasivayam Ambalavanan, and the LungMAP Consortium. LungMAP: the molecular atlas of lung development program. *American Journal of Physiology - Lung Cellular and Molecular Physiology*, 313:5 (2017) L733–L740. https://doi.org/10.1152/ajplung.00139.2017

**Value of the data**

• The data are a visual resource for the spatial distribution of expression for hundreds of key genes across a diversity of cell types in mouse lung development, providing a valuable tool for understanding the complex process of alveolarization and cellular differentiation, as well as spatial context and validation for quantitative transcriptome and proteome approaches.
• The data may assist in interpreting gene expression data in mouse models of disease and other experimental challenges.
• The associated mRNA probe sequences can assist researchers in generating their own probes, with the respective images providing validation for successful probe creation.
• The data may contribute to a better understanding of human lung development and disease.
• The data and method could form the foundation of a transcriptome approach to characterizing the spatial distribution of all differentially expressed genes in the mouse lung.

## 1 Data

**Fig. 1:**
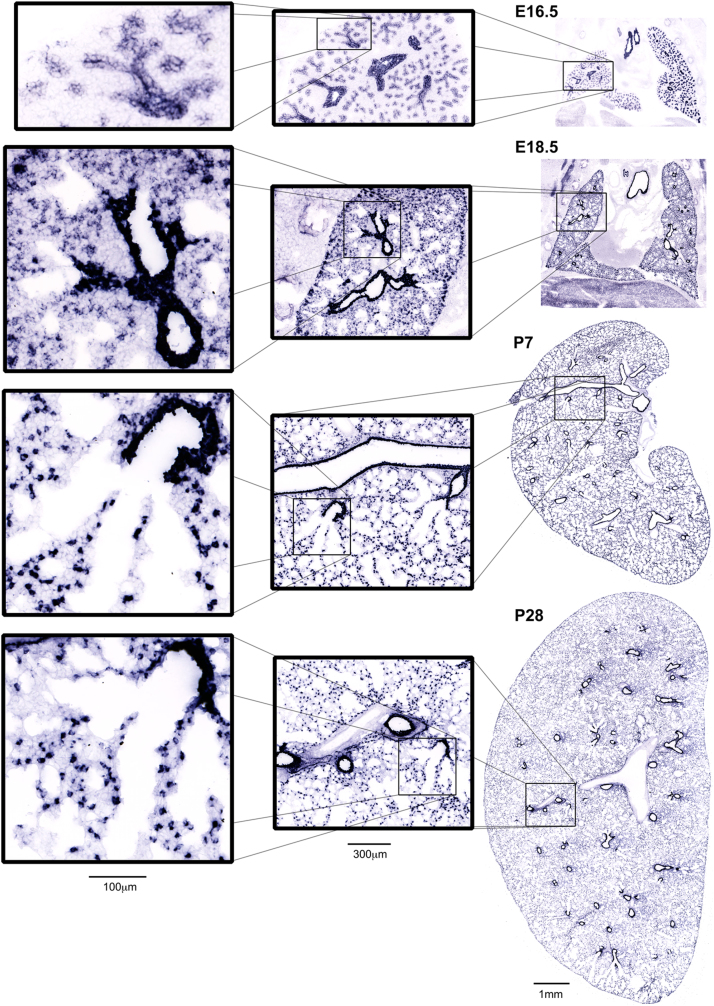
Illustration of HT-ISH image data for *Wfdc2* for one tissue section for each selected time point. Every tissue section is imaged in its entirety at sub-micron resolution and is presented here at the same scale. Scale bars apply to all sections.

The data consists of over 10,000 images representing the expression of 558 genes in the mouse lung across four key developmental time points. Images were generated using a high-throughput *in situ* hybridization (HT-ISH) approach that applies non-radioactive digoxigenin-labeled probes to thinly sliced frozen tissue sections. This is a systematic standardized approach that has been used across different organs and species [1], [2], [3], [4], [5], [6], [7], but not previously in mouse lung at this scale of production. For this work, cryosections were generated in the frontal plane. Time points were selected to represent canalicular to alveolar stages of lung development [8]. The canalicular stage is represented at embryonic day 16.5 (E16.5) and the saccular stage is represented at E18.5. Early alveolar stage is represented at postnatal day 7 (P7), and P28 represents late alveolar stage. The specific timepoints were selected based upon the initial recommendations made by the LungMAP Consortium [9]. In most cases, a minimum of four curated replicate images per gene and time point are included in the published dataset. An example of the data is displayed in Fig. 1.

The images are accessible at https://lungmap.net by selecting **in situ** under the **IMAGES** category, or by using the search feature to select a specific gene. Associated with the dataset are three tab-delimited XLS files: the first contains gene symbols, gene names, probe sequences, primer sequences, and direct URLs to genes and probes (GeneProbeList.xls, Supporting information); the second contains gene symbols and their single-cell RNA-Seq expression profiles clustered by time points and cell type (GenesByCellType.xls, Supporting information); and the third contains whole lung RNA-Seq expression levels over time by gene (GenesWholeLung.xls, Supporting information).

This data is part of an extensive effort to characterize the details of normal development of the lung during the key stages of alveolarization by collecting data across different modalities [9].

## 2 Experimental design, materials, and methods

Detailed Standard Operating Procedures (SOPs) are available at https://lungmap.net. All animal procedures were approved by the Institutional Animal Care and Use Committees (IACUC) at the respective institutions where live animals were handled.

### 2.1 Gene selection, probe design and generation

#### 2.1.1 Gene selection

Genes were selected to give a wide distribution of cell type/subtype specific markers and developmentally active genes, as informed by single cell RNA-Seq and whole-lung time course analysis.

**Fig. 2:**
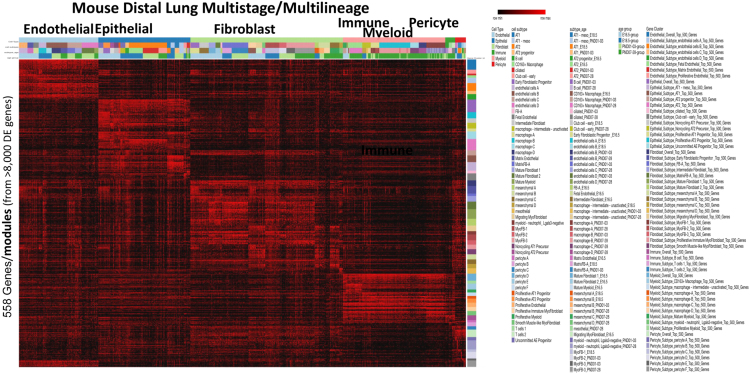
Expression levels of candidate marker genes based on LungMAP single cell RNA-Seq across 1000 cells are shown spanning development, differentiation, and physiology. Expression in log2(TPM+1) of each major class or subclass specific genes (rows) in each single cell (columns) are shown. Gene clusters were organized based on the cell population they belonged to and cells were organized based on their cell type, cell subtype, and developmental stage. With each gene and cell group, relative expression was used to reorder genes and cells, respectively. The heatmap was generated using Morpheus (software.broadinstitute.org/morpheus/).

Single cell RNA-Seq using Fluidigm C1 based microfluidics system was applied following the manufacturer׳s protocol to C57BL6/J mouse (Jackson Laboratory) whole lungs at E16.5, E18.5, P1, P3, P7, P10, P14, and P28. This provided a more detailed time coverage during saccular (P1-P3) and alveolar (P7-P28) development stages, which can shed light on any significant changes in expression within stages. Sequencing reads were aligned to mouse genome build GRCm38/mm10 and UCSC reference transcriptome using Tophat 2.0.9. Quantification was performed using Partek E/M quantification model [10] and normalized to transcripts per million (TPM) for downstream analysis. Criteria of Reads >500 and read quality > 30 were considered for sample QC inspection. Single cell data at individual time points were analyzed independently for cell type and signature gene prediction using SINCERA [11]. Differentially expressed (DE) genes of each cell population were calculated using Student׳s T-test between the target and reference populations, with p-value cut-off at 0.05. For major cell classes, the reference population was defined as all other cells not belonging to the target population, and the top 500 DE genes ranked descending by median log expression fold change (logFC) were used as the major class specific genes. For cell subclasses within a major cell class, the reference population was all other cells not belonging to the target cell subclass but within the parent major cell class. Each gene was then ranked based on major cell level and subclass level logFC descending, and the final rank was defined as the average of the two ranks. Fig. 2 summarizes this data for the 558 selected genes, and the data is available in the associated file GenesByCellType.xls, Supporting information.

Whole mouse lung gene expression profiling was performed by RNA-Seq on laser capture microdissected (LCM) lung tissue from mice in a time-course study to identify key developmental genes and time points. For this time-course study, C57BL/6 mouse (Jackson Laboratories) lungs were collected at E16.5, E18.5, and over the first 28 postnatal days of lung development (every 12 h for the first 14 d, then every 24 h) from mice euthanized with an overdose of isoflurane. Lungs were immediately embedded in Optimal Cutting Temperature compound (OCT) after dissection, snap frozen in liquid N_2_ and transferred to −80 °C storage. Tissue sections (16 µm) were cut using a cryostat and placed on onto membrane-glass slides (Arcturus PEN#LCM0522) at −25 °C. The OCT slides were washed and briefly dehydrated prior to LCM using Arcturus® HistoGene® LCM Frozen Section Staining Kit. Using the ArcturusXT system, lung parenchymal samples avoiding large bronchi or blood vessels were collected in CapSure® LCM Caps and stored in lysis buffer (Invitrogen) at −80 °C, and then used for further downstream analysis. Total RNA was extracted via miRneasy Micro Kit (Qiagen217084) according the manufacturer׳s protocol. After evaluation of RNA Integrity Number, 100 ng input were used for library preparation by Illumina® TruSeq® Stranded Total RNA Sample Preparation kits with Ribo-Zero according manufacturer׳s protocols. Sequencing was performed using an Illumina Hi-Seq. 2500 to generate paired-end 75 bp reads with a mean of 20 M reads per sample. Fastq files, after quality control for adapter contamination and trimming, were mapped on the mouse genome using STAR software [12]. The fragments per kilobase of transcript per million mapped reads (FPKM) matrix was generated by cufflinks. This data for the matching selected genes and time points is included in GenesWholeLung.xls, Supporting information.

#### 2.1.2 Probe design

The majority of the mRNA probes were identical to the mRNA probes published in www.genepaint.org [1], a small number were based on the mRNA probes published in www.brain-map.org [4], and the remaining were designed using sequence databases to be both unique to the gene investigated and 300–1000 bp long (optimal >600 bp). The probe accession numbers, template sequences, and primer sequences are available in GeneProbeList.xls, Supporting information.

#### 2.1.3 Probe generation

Digoxygenin-labeled mRNA probes were generated from DNA templates. The templates were generated by PCR from a cDNA pool, synthesized from RNA isolated from embryos at E14.5, as well as lung and brain at P7 and adult, using primers consisting of 20–25 nucleotides of gene-specific sequences linked to T3 or T7 polymerase promoter sequences. The gene specific template DNA (500 ng of DNA per reaction) was used for in vitro RNA synthesis in the presence of digoxigenin-tagged UTP using a kit from Roche. The concentration of the resulting DIG-labeled mRNA probes was quantified using a NanoDrop spectrophotometer and all probes were diluted to 100 ng/µl with ISH buffer and stored at −20 °C until use.

Probes were tested by ISH on E14.5 mouse sections and validated by comparison with published images [1], [4], or when image data was not available, by sequencing the DNA template.

### 2.2 Tissue collection and sectioning

#### 2.2.1 Tissue collection, embryonic tissue

C57BL/6J mice were purchased from the Jackson Laboratory and bred in-house according to standard breeding protocols. The day of vaginal plug was embryonic day 0.5. Embryos were harvested in the mornings of gestational day 16.5 or 18.5. The sex of each embryo was determined by collecting tail snips for PCR [13]. Each dissected embryo was trimmed by removing the head and the lower body below the liver, then placed in small cryomolds filled with OCT (Sakura/Fisher) and frozen on dry ice. Frozen blocks were kept at –80 °C until sectioned.

Whole embryos were collected at E14.5 to test the generated probes. The sex of E14.5 embryos was not recorded.

#### 2.2.2 Tissue collection, postnatal tissue

C57BL/6J mouse lungs were harvested on P7 or P28 after an overdose of ketamine/xylazine/acepromazine mixture. The lungs were inflated with a mixture of ice-cold OCT/PBS/sucrose solution through the trachea, and once removed from the chest cavity, dissected into the separate lobes and frozen in OCT. Frozen blocks were kept at –80 °C until sectioned. Notes were kept on sex of mice.

#### 2.2.3 Tissue sectioning

The fresh frozen embryo and postnatal specimens were sectioned at 20 µm on Leica CM3050S cryostats. Sections from each specimen were collected on Superfrost Plus slides (Fisher) and organized as 8 sets of slides with four sections per slide as previously detailed [14].

E16.5 and E18.5 sections were collected as intact torsos, ventral to dorsal. Postnatal lung sections were collected separately for different lobes, except sections for right upper and middle lobes, which were collected together.

E14.5 embryo sections for probe validation were collected as 25 µm sagittal sections, with only the sections including lungs collected, and organized as six sets with four sections per slide.

Following sectioning, the sections were allowed to air dry onto the slides at room temperature for a minimum of 30 min and then stored at –40 °C until fixation and acetylation. This step is described in detail in SOPs (https://www.lungmap.net/resources/sop-search-page/) and in [3]. After these steps, section quality was checked via microscope and the slides stored at –80 °C until use.

### 2.3 High-throughput in situ hybridization

We used HT-ISH to detect specific mRNA expression within sections of mouse lung using non-radioactive digoxigenin-labeled probes and chromogenic development of the signal. This approach uses several signal amplification steps, including tyramide. For each developmental time-point in mouse (E16.5, E18.5, P7, P28), two slides totaling eight sections were utilized for each mRNA probe during ISH.

Our HT-ISH protocol uses a Tecan liquid-handling robot with GenePaint technology described in [7]. Briefly, slides carrying tissue sections were inserted into flow-through chambers, which were then placed into a temperature-controlled rack positioned onto a Tecan EVO GenePaint liquid handling platform. All solutions were added using a computer controlled liquid handling system. The liquid handling system software also regulates the water circulator baths that control the temperature of both the slide racks and the solutions. The proteinase K concentration, but not incubation time, was modified for different developmental stages (2 µg/ml E14.5, E16.5 and E18.5 tissue, 4 µg/ml P7 tissue, 7 µg/ml P28 tissue). We used a standardized probe concentration during hybridization of 300 ng/ml. We found this to be a suitable concentration for more than 95% of the genes. Exceptions were when the staining was either very weak at all time-points, in which case the probe concentration was increased to 600 ng/ml, or in the rare instances when the gene expression was exceptionally high at all time-points, then the probe concentration was reduced to 150 ng/ml.

Following HT-ISH, the slides were removed from the machine, rinsed in water and cover-slipped in Hydro-Matrix mounting medium (Micro Tech Lab, Austria) using a Leica CV5030 automatic cover-slipper.

### 2.4 Imaging and image curation

#### 2.4.1 Imaging

All slides from HT-ISH were imaged using a Zeiss Axioscan.Z1 whole slide scanner. The slides were imaged using a 20×/0.8 lens (0.22 µm/pixel resolution) and whole section images were stitched together by the Zeiss Zen software [15]. Images were exported from Zen at 25% size in PNG format, resulting in images at 0.88 µm/pixel resolution.

#### 2.4.2 Curating images for public dissemination

Images were stored on CyVerse [16] and the Bisque interface [17] was used to facilitate distributed curation. The eight tissue sections per probe per time point were individually ranked based on an overall ability of the image to accurately illustrate the gene expression in the lung. This overall subjective determination was a combination of ISH expression signal quality (selectivity and uniformity), tissue quality (minimal tears, folds), and minimal artifacts (dust, air bubbles). The top four imaged sections from each group of eight were used to define the minimum standard of quality for images to be made public.

For P7 and P28, four images were from left lung, and four images from one of the lobes from the right lung. At these ages, the standard of image quality was tracked independently between left and right lung, with a minimum of two images from each selected to be made public.

In addition, for each time point for each probe, a representative image was selected to be highlighted. This representative image is the image that best illustrates the variety of anatomical features found in the left lung while also meeting the minimum standard of quality.

All curation determinations were reviewed by an expert histologist.

#### 2.4.3 Exceptions

In some rare cases, fewer than the desired minimum number of sections met basic expectations of quality. However, if fewer than two images for a specific probe at a given age met minimal expectations of quality, the entire experiment for all ages for that probe was repeated on a new set of tissue sections.

#### 2.4.4 Distribution

Image metadata and images were transferred to the LungMAP Data Coordinating Center (DCC) for distribution to the scientific community. Processing steps carried out at the LungMAP DCC included: rotation of images, where necessary using ImageMagick® (www.imagemagick.org); generation of image thumbnails using ImageMagick; generation of image tiles at multiple zoom levels using gdal2tiles.py [18] to enable display of images in the LungMAP image viewer and OpenLayers-based (www.openlayers.org) annotation tool; preparation of archives containing all images for a given age/probe combination and protocols for data collection; and storage of images and their derivatives in the Amazon Web Services Simple Storage Service. Image metadata and pointers to the images and their derivatives were stored in the LungMAP OpenLink Virtuoso triplestore database (https://virtuoso.openlinksw.com). Images were published on the LungMAP website (https://www.lungmap.net) [9]. Direct URLs for each gene and probe are included in GeneProbeList.xls, Supporting information.

## Footnotes

Appendix A Supplementary data associated with this article can be found in the online version at https://doi.org/10.1016/j.dib.2018.10.150.

## References

[1] Visel A. (2004). GenePaint.org: an atlas of gene expression patterns in the mouse embryo. Nucleic Acids Res..

[2] Carson J.P. (2005). A digital atlas to characterize the mouse brain transcriptome. PLoS Comput. Biol..

[3] Yaylaoglu M.B. (2005). Comprehensive expression atlas of fibroblast growth factors and their receptors generated by a novel robotic in situ hybridization platform. Dev. Dyn..

[4] Lein E.S. (2007). Genome-wide atlas of gene expression in the adult mouse brain. Nature.

[5] Visel A. (2007). Regulatory pathway analysis by high-throughput in situ hybridization. PLoS Genet..

[6] Shcherbatyy V. (2015). A digital atlas of ion channel expression patterns in the two-week-old rat brain. Neuroinformatics.

[7] Geffers L., Hauptmann G. (2015). High-throughput In Situ hybridization: systematical production of gene expression data and beyond. In Situ Hybridization Methods.

[8] Warburton D. (2010). Lung organogenesis.

[9] Ardini-Poleske M.E. (2017). LungMAP: the molecular atlas of lung development program. Am. J. Physiol. - Lung Cell. Mol. Physiol..

[10] Xing Y. (2006). An expectation-maximization algorithm for probabilistic reconstructions of full-length isoforms from splice graphs. Nucleic acids Res..

[11] Guo M. (2015). SINCERA: a pipeline for single-cell RNA-Seq profiling analysis. PLoS Comput. Biol..

[12] Dobin A. (2015). Mapping RNA-seq Reads with STAR. Curr. Protoc. Bioinforma..

[13] Clapcote S.J. (2005). Simplex PCR assay for sex determination in mice. BioTechniques.

[14] Carson J.P. (2010). Automated pipeline for atlas-based annotation of gene expression patterns: application to postnatal day 7 mouse brain. Methods.

[15] ZEISS ZEN – Digital Imaging for Light Microscopy. 〈https://www.zeiss.com/microscopy/int/products/microscope-software/zen.html〉, 2018 (accessed 24.09.18).

[16] Merchant N. (2016). The iPlant Collaborative: cyberinfrastructure for enabling data to discovery for the life sciences. PLoS Biol..

[17] Kvilekval K. (2010). Bisque: a platform for bioimage analysis and management. Bioinformatics.

[18] GDAL/OGR Geospatial Data Abstraction Library. 〈https://www.gdal.org/〉, 2018 (accessed 24.09.18).

